# Distribution and diversity of myxomycetes in Tiantangzhai National Forest Park, China

**DOI:** 10.7717/peerj.12059

**Published:** 2021-08-25

**Authors:** Min Li, Gaowei Wang, Yang Gao, Mingzhu Dou, Ziqi Wang, Shuzhen Yan, Shuanglin Chen

**Affiliations:** 1 College of Life Sciences, Nanjing Normal University, Nanjing, China; 2 Henan Key Laboratory of Children’s Genetics and Metabolic Diseases, Children’s Hospital Affiliated to Zhengzhou University, Henan Children’s Hospital, Zhengzhou Children’s Hospital, Zhengzhou, China; 3 Bioengineering and Technological Research Centre for Edible and Medicinal Fungi, Jiangxi Agricultural University, Nanchang, China

**Keywords:** Plasmodial slime molds, Subtropics, Forest type, Season

## Abstract

Although myxomycetes are ubiquitous in terrestrial ecosystems, studies on their distribution and diversity in subtropical humid forests are still lacking. Field collections and moist chamber cultures were conducted from May to October within a two-year period in the Tiantangzhai National Forest Park of China. A total of 1,492 records representing 73 species belonging to 26 genera were obtained, of which 243 records/37 species were from field collections, and 1,249 records/52 species were from moist chamber cultures. Among the specimens obtained by culturing, 896 records/38 species and 353 records/37 species were obtained from living bark and ground litter, respectively. ANOVA showed that the sampling months had significant impacts on collection of myxomycetes from field and those that inhabit litter. An LEfSe analysis indicated that *Arcyria* was significantly abundant in August, while *Stemonitis* and *Physarum* were more abundant in July when collected from field. An RDA analysis showed that temperature was the main factor that affected the litter-inhabiting myxomycetes. The ANOVA indicated that forest type was the significant factor for bark-inhabiting myxomycetes. *Diderma effusum* was primarily obtained from mixed forests, while *Clastoderma debaryanum* and *Colloderma oculatum* were more common in coniferous forests. The RDA analysis indicated that the vegetation, pH, water retention, and elevation were the primary factors that affected the bark-inhabiting myxomycetes.

## Introduction

Myxomycetes are a group of eukaryotic microorganisms in terrestrial ecosystems, which are common in bark, woody debris, litter, lianas, bryophytes, dung, and soil (Liu, Yan & Chen, 2015). The life cycle of myxomycetes is composed of two trophic stages including uninucleate myxamoebae or flagellate cells and multinucleate plasmodium and one sporulating stage (fruiting bodies). In addition, the myxamoebae and plasmodium are transformed into microcysts and sclerotia, respectively, under unfavorable conditions, which are called propagules together with spores. The plasmodia feed on bacteria, yeasts, algae, and other microorganisms, which can regulate the microbial community and affect the distribution of nutrition and biomass in microhabitats (Dagamac, Stephenson & Dela Cruz, 2014; Zahn, Wagai & Yonemura, 2016; Hunninghaus et al., 2017).

Myxomycetes are widely distributed around the world, and approximately 1,000 morphospecies have been observed (Lado, 2005–2020). They are currently recorded in tropical, subtropical, temperate, boreal forests, tundra, grasslands, deserts, and alpine snowbanks (Schnittler, Dagamac & Novozhilov, 2017). However, in terms of the distribution of the world’s climatic zones, the distribution in tropical and temperate climates is far more extensive than that of subtropical humid climates, and the species of myxomycetes in subtropical forests have not been sufficiently studied. Subtropical regions have a humid climate and diverse vegetation, and they are located in the transition region between tropics and temperate zone. Therefore, they should have abundant resources of myxomycete species. Researchers in Vietnam (Redeña-Santos et al., 2018), Nepal (Chauhan, Budhathoki & Adhikari, 2015), China (Liu et al., 2013; Gao et al., 2018; Ukkola, Härkönen & Zeng, 2001; Härkönen, Ukkola & Zeng, 2004) and other countries have conducted research on the species of humid subtropical myxomycetes. A total of 239 species of myxomycetes belonging to 41 genera have been identified in subtropical China. Therefore, this region is of substantial significance for the investigation and diversity research on myxomycetes species in subtropical areas.

The main influencing factors of species diversity of myxomycetes are divided into abiotic factors, such as temperature, humidity, altitude, water retention and pH, and biological factors, such as vegetation type, bacterial composition, fungal composition (Xavier de Lima & de Holanda Cavalcanti, 2015; Liu, Yan & Chen, 2015). The distribution of vegetation has the characteristics of vertical zoning, demonstrating the replacement of plant communities as the altitude increases. For example, the altitude of coniferous forests in naturally formed forests is generally higher than that of broad-leaved forests. The species assemblage of myxomycetes varies in different forest types, which generally indicate that the abundance of myxomycetes in deciduous broad-leaved forests is significantly higher than that in coniferous forests (Takahashi & Hada, 2012; Novozhilov et al., 2017). Some species have a preference for the type of vegetation. For example, *Physarum* and *Didymium* are more common in tropical and subtropical broad-leaved forests, while *Cribraria* and *Trichia* are widely distributed in temperate coniferous forests (Stephenson, Novozhilov & Schnittler, 2000). Several factors that affect the occurrence of myxomycetes in forest types include the substrate, pH, water retention, and vegetation (Schnittler et al., 2016; Gao et al., 2018).

The occurrence of myxomycetes shows some seasonal regularity. For example, the results show that in Virginia, USA, the species richness and diversity of myxomycetes peaked in August and gradually declined during the following months (Stephenson, 1988). Myxomycete fruiting bodies were significantly higher during the rainy season than dry season in low-altitude forests and farmland in Thailand, but there were more propagules, including spores, microcysts, and sclerotia, on the substrate collected during dry season (Ko Ko et al., 2011; Tran et al., 2006; Tran et al., 2008). Subtropical regions are ideal places to study the seasonal regularity of myxomycetes because of their distinct characteristics of temperature and precipitation seasons.

In this study, fruiting bodies in field, as well as living bark and ground litter for moist chamber cultures were collected monthly in three forest types from May to October in 2015 and 2016 in the Tiantangzhai National Forest Park, a subtropical region of China. The main purpose of this study through field collections and moist chamber cultures in this area was to: (i) describe the species composition of myxomycetes; (ii) explore the influence of forest types and months on the richness and diversity of myxomycetes, and (iii) evaluate the relationship between environmental factors and myxomycete communities, thereby further supplementing the data of myxomycetes in subtropical forests.

## Materials & Methods

### Study area

**Figure 1:**
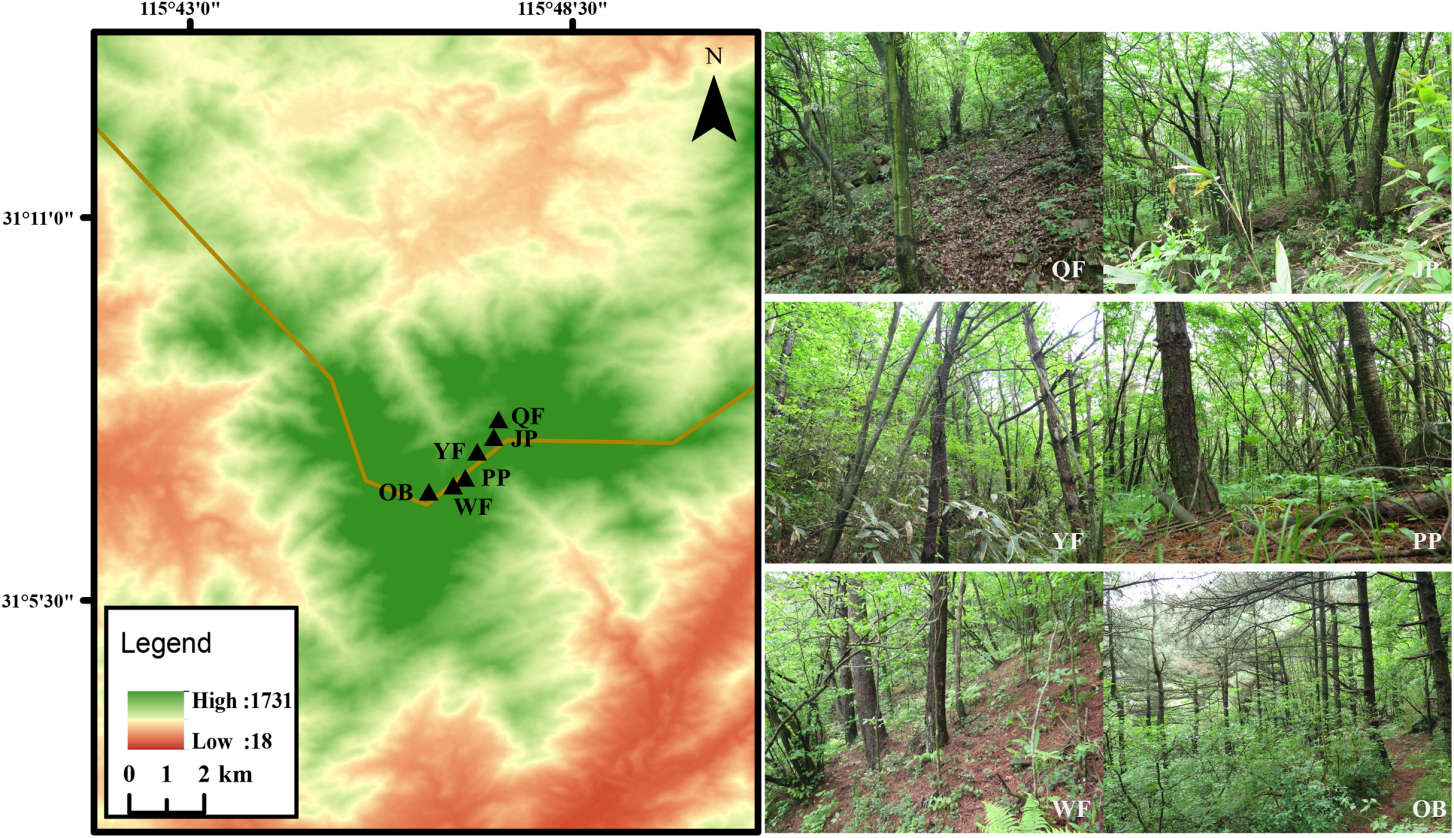
Map of six study sites in Tiantangzhai National Forest Park, Anhui Province, China.

The Tiantangzhai National Forest Park (31°06′–31°10′N, 115°45′–115°48′E) is located in Jinzhai County, Anhui Province, China (Fig. 1). The forest park belongs to the north subtropical warm humid monsoon climate, with an annual average temperature of 14.6 °C and a maximum average temperature of 27.9 °C. The frost-free period is approximately 220 days, and the annual rainfall reaches 1,280–1,300 mm a.s.l. The broad-leaved forest is dominated by *Cyclobalanopsis myrsinifolia*, *Quercus aliena* var. *acuteserrata*, *Q. serrata* var. *brevipetiolata*, and many shrubs. The obvious vertical distribution of forest is that there are more *Pinus taiwanensis* distributed over 1,300 m a.s.l. with the increase in elevation.

### Study sites

Six study sites (50 m × 50 m) were selected in the 1,000–1,600 m a.s.l. altitude range of the Tiantangzhai National Forest Park, with an interval of approximately 100 m a.s.l. between adjacent sites. These six study sites were close to rivers that had little disturbance from humans, and had the main tree species of the forest park. The specific location and vegetation information are as follows:

Qingren Fall (abbr. QF; 31°7′8″N, 115°47′21″E; 1,035 m a.s.l.) is a deciduous broad-leaved forest, and the main trees are *Cyclobalanopsis myrsinifolia*, *Q. aliena* var. *acuteserrata*, and *Castanea seguinii*.

Jade Pool (abbr. JP; 31°7′48″N, 115°47′19″E; 1,168 m a.s.l.) is a deciduous broad-leaved forest, and the main trees are *Q. serrata* var. *brevipetiolata*, *Cercis chinensis*, *Q. aliena* var. *acuteserrata*.

Yingong Fall (abbr. YF; 31°7′38″N, 115°47′7″E; 1,294 m a.s.l.) is a coniferous broad-leaved mixed forest, and the main trees are *Pinus taiwanensis*, *Q. serrata* var. *brevipetiolata*, and *Platycarya strobilacea*.

Pheasant Plain (abbr. PP; 31°7′15″N, 115°46′57″E; 1,405 m a.s.l.) is a coniferous broad-leaved mixed forest, and the main trees are *P. taiwanensis*, *C. seguinii*, and *Euptelea pleiospermum*.

Wild boar Forest (abbr. WF; 31°7′15″N, 115°46′57″E; 1,532 m a.s.l.) is a coniferous forest, and the main trees are *P. taiwanensis* and *Lindera obtusiloba*.

The Observatory (abbr. OB; 31°7′6″N, 115°46′33″E; 1,600 m a.s.l.) is a coniferous forest, and the main trees are *P. taiwanensis* and *Indocalamus tessellatus*.

### Sampling

A total of 12-time surveys, including the collection of fruiting specimens and substrates, were performed monthly in the study sites from May to October in 2015 and 2016. The myxomycete fruiting bodies were carefully searched within the study sites and placed in labeled sample boxes. The specimens were brought back to the laboratory and air-dried for storage. In each study site, the diagonal method was used to select five plots (10 m × 10 m) at the four corners and center point. The bark of living trees that were randomly selected from each plot at a height of 1 m and the ground litter, such as dead branches and fallen leaves under the trees, were collected as substrates. A total of 720 substrate samples were collected.

### Moist chamber cultures

Moist chamber cultures of living bark and ground litter collected from the different six study sites were prepared as described by Stephenson & Stempen (1994). Three moist chambers were prepared for each individual substrate. The pH of each dish was measured using a flat plate pH meter (Fisher Scientific Accumet Model PHS-3C; JingKe Company, Shanghai, China). The method to determine water retention of the substrate was that used by Snell & Keller (2003).

### Taxonomy

The fruiting bodies were primarily identified based on their morphological characteristics (Martin & Alexopoulos, 1969). The nomenclature followed Lado (2005–2020). Ceratiomyxales, the traditional research subject of myxomycetologists, has also been included in the study. Specimens were deposited in the Microbial Culture Center of Nanjing Normal University (MCCNNU), Nanjing, China.

### Data analysis

The same species in the same Petri dish in the moist chamber culture was recorded as one collection. The relative abundance dataset of myxomycete species was subdivided into field collections (FC), moist chamber cultures-bark (MC-bark), and moist chamber cultures-litter (MC-litter) assemblages. The relative abundance of each species was obtained by dividing the number of records for each species of myxomycetes by the total number of myxomycetes (Stephenson, Kalyanasundaram & Lakhanpal, 1993). The species abundance scale was classified as follows: A: abundant (>3%); C: common (1.5–3%); O: occasional (0.5–1.5%); R: rare (<0.5%). To evaluate the sampling completeness of these assemblages, EstimateS 9.1.0 was used to calculate the Chao1 estimator based on the number of records (Chao et al., 2005). Shared species of three forest types and six sampling months were plotted using the R-package “VennDiagram” (Chen, 2018) and “venn” (Dusa, 2020). Origin 2017 (OriginLab, Northampton, MA, USA) was used to draw a bidirectional bar chart to describe the relative abundance of >1% species.

The number of species (*S*) and Shannon–Wiener diversity index (*H′*) were used to assess the richness and diversity of myxomycetes. An analysis of Variance (ANOVA) and a Fisher’s protected least significant difference (LSD) or a Kruskal–Wallis test was used to compare the differences in species richness and diversity of FC, MC-bark, and MC-litter assemblages in different sites, forest types, and months. These steps were performed in SPSS 25 (IBM, Inc., Armonk, NY, USA) and Origin 2017.

A cluster analysis based on Bray–Curtis distance further revealed the ecological model of myxomycete communities in assemblages (FC-month, MC-bark-forest type, and MC-litter-month) that showed significant differences in the previous ANOVA analysis. ANOSIM was used to test significant differences between the groups. To further and specifically identify myxomycete taxa associated with forest types and sampling months, linear discriminate analysis effect size (LEfSe) was used to compare the relative abundance of species at different taxonomic levels. Several myxomycete taxa were found to be differentially abundant between the study groups using a linear discriminant analysis (LDA) score > 4.0 and *P* < 0.05. These cases were analyzed using the metaMDS and anosim within the R-package “vegan” (Oksanen et al., 2018), and an online resource (http://huttenhower.sph.harvard.edu/galaxy).

The influence of environmental factors on the myxomycete community of MC-bark and MC-litter was analyzed by redundancy analysis (RDA) using the R-package “vegan” (Oksanen et al., 2018). To avoid interference from rare species, individual observations in the species abundance matrix were removed, and the relative abundance retained was converted logarithmically. Environmental factors included temperature, precipitation, elevation, season, pH, and water retention of the substrates. The ordir2step within the “vegan” was performed to carry out positive analysis for each variable using 999 times of a permutation test. The temperature and precipitation data originated from the Anhui Meteorological Observatory of China, Hefei, China.

## Results

### Species composition

A total of 1,492 records were obtained from field collections (FC) and moist chamber cultures (MC-bark and MC-litter), representing 73 species belonging to 26 genera. A total of 243 records of myxomycetes were collected from the field, belonging to 37 species and 17 genera. A total of 1,249 records belonging to 52 species and 21 genera were obtained from the moist chamber cultures. There were 896 myxomycete specimens on the bark belonging to 38 species and 353 on the litter belonging to 37 species. The distribution of species at the order level was as follows: Physarales (22 species), Trichiales (17 species), Liceales (17 species), Stemonitidales (14 species), Echinosteliales (2 species), and Ceratiomyxales (one species). Based on their relative abundance, seven species were recorded as A (abundant), including *Arcyria cinerea*, *Clastoderma debaryanum*, *Colloderma oculatum*, *Diderma effusum*, *Echinostelium minutum*, *Licea operculata*, and *Lycogala exiguum*. In addition, 12 species were recorded as C (common), 14 as O (occasional) and 40 as R (rare). Only nine species, including *A. cinerea*, *Arcyria denudata*, *Comatricha nigra*, *Cribraria microcarpa*, *D. effusum*, *Hemitrichia serpula*, *Physarum album*, *Stemonitis fusca*, and *Trichia botrytis*, were found in the FC, MC-bark, and MC-litter. A total of 21 species, including *Amaurochaete atra*, *L. exiguum*, *Reticularia lycoperdon*, and *Tubifera ferruginosa*, only appeared in the FC. Fourteen species, including *C. oculatum*, *Cribraria confusa*, and *Macbrideola scintillans*, were found only on the bark. Eight species, including *Diachea subsessilis*, *Physarum bivalve*, and *Physarum lateritium*, were specific for litter (Table 1; Data S1).

**Table 1:** Myxomycete composition and relative abundance from experimental methods, forest types and sampling months.

Species name	Abbreviation	Overall^a^	Experimental methods^b^			Forest types^c^			Sampling months					
			FC	MC-Bark	MC-Litter	DBF	CBMF	CF	May	Jun	Jul	Aug	Sep	Oct
*Amaurochaete atra*	AMAatr	R	R				R						R	
*Arcyria cinerea*	ARCcin	A	A	A	A	A	A	A	C	A	A	C	O	O
*Arcyria denudata*	ARCden	C	O	R	C	O	O	O	R	O	O	O	R	R
*Arcyria major*	ARCmaj	R	R			R						R		
*Arcyria obvelata*	ARCobv	O	O			R	R	R			C			
*Calomyxa metallica*	CALmet	R		R	R	R	R		O		R	R		
*Ceratiomyxa fruticulosa*	CERfru	C	C		R	R	O	O	O	O	C	R	O	R
*Clastoderma debaryanum*	CLAdeb	A		A	C	O	A	A	C	O	C	C	O	C
*Collaria arcyrionema*	COLarc	O	O		R	R	R	R			O	O		O
*Colloderma oculatum*	COLocu	A		A			A	A	C	C	C	C	C	O
*Comatricha nigra*	COMnig	O	R	R	R	R	O	R	R	O		R		
*Comatricha pulchella*	COMpul	R		R	R		R	R		R	R	R	R	
*Cribraria aurantiaca*	CRIaur	C	C			R	C	O		O	O	O		
*Cribraria confusa*	CRIcon	O		O		O			O	R	O	R		
*Cribraria microcarpa*	CRImic	C	O	O	R	R	O	O	R		O	O	R	R
*Cribraria minutissima*	CRImin	R		R			R	O	R	R		O		
*Cribraria violacea*	CRIvio	R	R		R	O	R		O	O	R			
*Diachea splendens*	DIAspl	R			R	O								O
*Diachea subsessilis*	DIAsub	R			R	O	O					O		O
*Dictydium cancellatum*	DICcan	C	C			R	C	R			C	R		
*Diderma effusum*	DIDeff	A	O	C	O	R	C	O	R	O	O	O	R	O
*Diderma hemisphaericum*	DIDhem	R	R		R	O		R				R		O
*Didymium clavus*	DIDcla	R		R		O						O		
*Didymium iridis*	DIDiri	R	R				R					R		
*Didymium melanospermum*	DIDmel	R		R		O							O	
*Didymium minus*	DIDmin	O	O	R		O	R	R		O	R			
*Didymium squamulosum*	DIDsqu	R		R		O	O		O	O				
*Echinostelium minutum*	ECHmin	A		A	O	O	C	C	O	O	C	O	R	O
*Hemitrichia calyculata*	HEMcal	C	C			R	C	R		R	C	O		
*Hemitrichia minor*	HEMmin	R		R	R	O	R					O	R	
*Hemitrichia serpula*	HEMser	O	O	R	R	R	O	R	R	O	R	R		R
*Licea biforis*	LICbif	R		R		O	R		O		O	O		R
*Licea castanea*	LICcas	R		R	R	R	O		R			O	R	R
*Licea kleistobolus*	LICkle	R			R		O			O				
*Licea minima*	LICmin	O		O	R	R	O	R	R	O	R	R	R	R
*Licea operculata*	LICope	A		A	R	R	C	O	R	R	O	O	O	O
*Licea pedicellata*	LICped	R		R		R				O	O	R		
*Licea pusilla*	LICpus	R		R		O	O	O				R	O	
*Licea variabilis*	LICvar	R		R	R			R		O				O
*Lycogala exiguum*	LYCexi	A	A			C	C	O	C	O	C	R	R	
*Macbrideola scintillans*	MACsci	R		R		R			R	O		R		
*Paradiacheopsis erythropodia*	PARery	O		O	R	R	R	R	R	O	R	R	O	
*Paradiacheopsis fimbriata*	PARfim	R		R		O	O					R		
*Perichaena corticalis*	PERcor	C		O	O	O	C	R	R	R	O	R	R	R
*Perichaena depressa*	PERdep	R	R		R	O	R				R		O	
*Perichaena pedata*	PERped	R		R			O			O				
*Physarum album*	PHYalb	C	O	C	R	O	O	O	R1	R	O	R	R	O
*Physarum bivalve*	PHYbiv	R			R	O			O					
*Physarum cinereum*	PHYcin	R			R		O				O			
*Physarum flavicomum*	PHYfla	O	O			R	R				O			
*Physarum globuliferum*	PHYglo	R	R					R			R			
*Physarum lateritium*	PHYlat	R			R	O								O
*Physarum melleum*	PHYmel	R	R		R	R	R		O		R	R		
*Physarum nucleatum*	PHYmus	R			R	O					O			
*Physarum oblatum*	PHYobl	R		R				O				O		
*Physarum penetrale*	PHYpen	O	O					O			O			
*Physarum pusillum*	PHYpus	R		R	R	R	O	R		R	R	O	O	O
*Physarum roseum*	PHYros	R	R			R						R		
*Physarum viride*	PHYvir	C	C			R	O	O			C			
*Reticularia lycoperdon*	RETlyc	R	R					R			R			
*Stemonitis axifera*	STEaxi	O	O			O	R	R			O	R		
*Stemonitis fusca*	STEfus	C	O	R	C	O	O	O	R	R	O	O	R	O
*Stemonitis pallida*	STEpal	R	R				R				R			
*Stemonitis smithii*	STEsmi	C	C			C	O	R			C			
*Stemonitopsis aequalis*	STEaeq	R		R			O					O		
*Stemonitopsis reticulata*	STEret	O		R	O	O	O	R	R	O	R	R	O	R
*Trichia botrytis*	TRIbot	C	R	O	R	O	R	O	R	R	R	O	O	O
*Trichia contorta*	TRIcon	R	R					R				R		
*Trichia decipiens*	TRIdec	O			R			O	O					
*Trichia erecta*	TRIere	R	R					R			R			
*Trichia favoginea*	TRIfav	R		R	R	R			O	R		O		
*Trichia lutescens*	TRIlut	R	R				R	R				R		R
*Tubifera ferruginosa*	TUBfer	O	O			R	R				O			
Number of species		73	37	38	37	54	52	42	31	33	45	48	25	26

**Notes:**

a Relative abundance of records for a particular species (Stephenson, Kalyanasundaram & Lakhanpal, 1993). A, abundant (>3% of all records); C, common (1.5–3%); O, occasional (0.5–1.5%); R, rare (<0.5%).

b Experimental methods include field collections (FC) and moist chamber cultures (MC-bark and MC-litter).

c Forest types include deciduous broad-leaved forest (DBF), coniferous broad-leaved mixed forest (CBMF), and coniferous forest (CF).

### Sampling completeness of the survey

**Figure 2:**
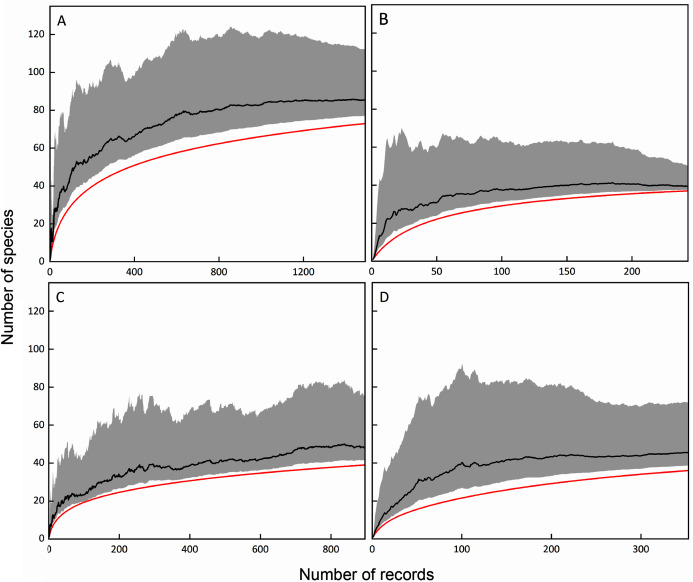
Species accumulation curves (red lines) and Chao1 estimators (black lines) with lower and upper 95% confidence intervals (grey shades); (A) total assemblage; (B) FC; (C) MC-bark; (D) MC-litter.

The Chao1 estimators were calculated based on the number of species and records of total, FC, MC-bark, and MC-litter myxomycete assemblages. Based on the results, if the sampling was thorough, the number of myxomycete species in the total, FC, MC-bark, and MC-litter assemblages could be expected to be calculated as 85, 39, 48 and 45, respectively. Correspondingly, the sampling completeness of these four assemblages was 86%, 95%, 79%, and 82%, respectively (Fig. 2).

### Spatial and temporal distribution of myxomycetes

**Figure 3:**
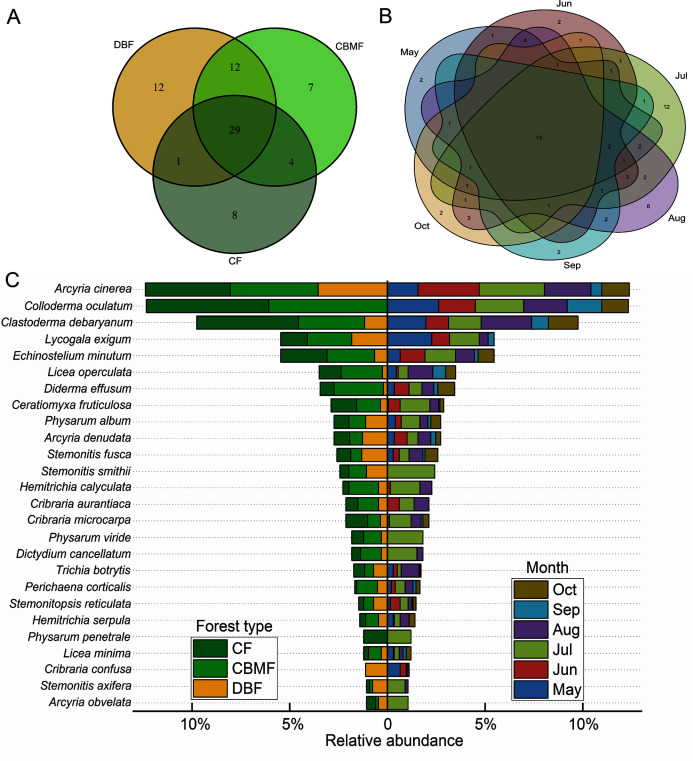
Venn diagrams of the myxomycete communities of (A) three forest types and (B) six sampling months and (C) the bidirectional bar chart of species distribution with relative abundance >1%. (DBF, deciduous broadleaf forest; CBMF, mixed coniferous and broadleaf forest; CF, coniferous forest).

Among the three forest types in the Tiantangzhai National Forest Park, 54 species of myxomycetes were obtained in the deciduous broad-leaved forest (DBF), 52 species in the coniferous broad-leaved mixed forest (CBMF) and 42 species in the coniferous forest (CF). In the CBMF, 41 species were shared with the DBF, and 33 were shared with the CF. The DBF and CF shared 30 species. A total of 29 species were distributed in all three forest types (Fig. 3A). From May to October during the two years as a whole, the distribution of myxomycete species increased and then declined, with 31, 33, 45, 48, 25, and 26 species obtained, respectively. Fifteen species were found in all the sampling months (Fig. 3B). The distribution of species with a relative abundance >1% in different forest types and months is shown by the bidirectional bar chart (Fig. 3C). What was more notable was that *Physarum penetrale* only occurred in the CF, and *C. confusa* only occurred in the DBF. Twelve species were only recorded in July within six sampling months, including *Arcyria obvelata*, *Physarum viride*, *P*. *penetrale*, and *Stemonitis smithii* (Data S2).

### Species diversity

**Figure 4:**
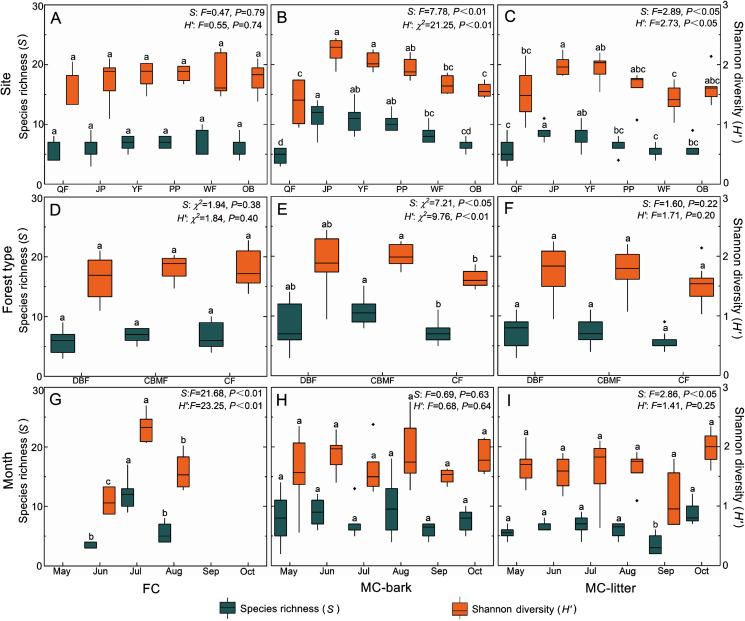
Box plots of species richness (green, left scale) and Shannon diversity (orange, right scale) for (A, D, and G) FC, (B, E, and H) MC-bark and (C, F, and I) MC-litter in different study sites, forest types and months. The ANOVA results are shown on the upper right. Different lowercase letters indicate significant differences (*P* < 0.05).

The changes in species richness and Shannon diversity of myxomycetes in the three assemblages of FC, MC-bark, and MC-litter in the six study sites are shown in Figs. 4A–4C. The species richness and Shannon diversity of myxomycetes in the MC-bark (*S*: *P* < 0.001, *H′*: *P* < 0.001) and MC-litter (*S*: *P* = 0.04, *H′*: *P* = 0.04) differed significantly from study site to study site but not in the FC (*S*: *P* = 0.79, *H′*: *P* = 0.74). In the MC-bark and MC-litter, the study site with the most species richness and Shannon diversity was JP (Figs. 4B, 4C; Data S3). In the MC-bark assemblage, the richness and diversity indices were lowest in the QF study site. The study site with lowest species richness was QF, while the lowest Shannon diversity was WF, which was in the MC-litter assemblages. In FC, the study site PP had the highest and the study site QF had the lowest points of myxomycetes species richness and Shannon diversity, respectively.

The richness and diversity of species varied significantly between forest types for the MC-bark assemblage (*S*: *P* = 0.03, *H′*: *P* = 0.01; Fig. 4E), but not between the forest types for FC (*S*: *P* = 0.38, *H′*: *P* = 0.40; Fig. 4D) and MC-litter assemblages (*S*: *P* = 0.22, *H′*: *P* = 0.20; Fig. 4F). The forest type with the lowest species richness and Shannon diversity in MC-bark and MC-litter was the CF, but the lowest in FC assemblage was DBF. The forest type with the highest value was recorded in the CBMF in FC and MC-bark, while the highest for MC-litter was recorded in the DBF.

Although specimens of myxomycetes were obtained in each sampling month, the richness and diversity analysis of myxomycetes could not be performed in the FC assemblage in May, September, and October owing to the scarcity of specimens. Seasonal fluctuations of species richness and Shannon diversity showed different patterns in the FC and MC-bark and MC-litter. The most abundant specimens could be obtained in the field in July (Fig. 4G) but not in the MC-bark and MC-litter. August was found to have the highest myxomycete species richness and Shannon diversity in the MC-bark assemblage (Fig. 4H), while those of the MC-litter were found in October (Fig. 4I). September had the lowest number of myxomycete species in the MC-bark and MC-litter.

### Analysis of the myxomycete community

**Figure 5:**
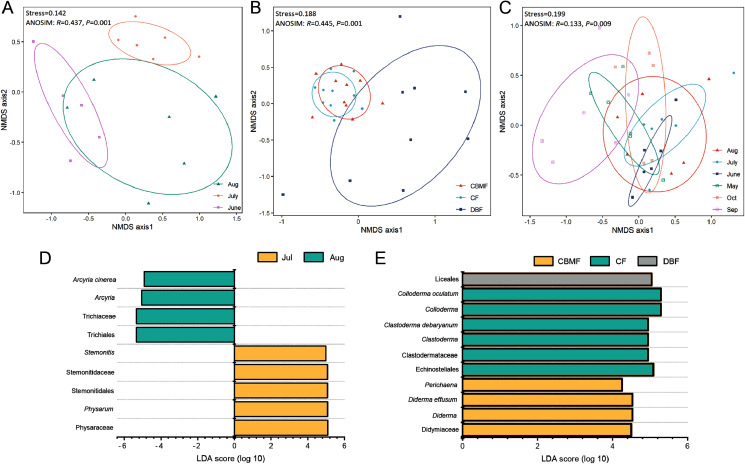
Non-metric multidimensional scaling built on Bray–Curtis distance and LEfSe analysis of (A, D) FC, (B, E) MC-bark and (C) MC-litter.

NMDS (Data S4–S6) combined with ANOSIM (Data S7) and LEfSe (Data S8–S10) further revealed the ecological model of the FC, MC-bark, and MC-litter myxomycete communities, which had significant differences according to the ANOVA analysis described above. The FC assemblage was clustered into three groups based on the month (Stress = 0.142; Fig. 5A). ANOSIM confirmed the significant difference among the three months (*R* = 0.437, *P* = 0.001). LEfSe further identified that *Arcyria* was significantly enriched in August, while *Stemonitis* and *Physarum* were preferentially more abundant in July (Fig. 5D).

NMDS ordination of the MC-bark showed three significantly different groups by forest type (Stress = 0.188; ANOSIM: *R* = 0.445, *P* = 0.001; Fig. 5B). The LEfSe showed that *Clastoderma* and *Colloderma* were significantly enriched in the CF and Liceales in the DBF, while *Diderma* and *Perichaena* were more abundant in the CBMF (Fig. 5E).

The MC-litter assemblage was divided into six groups by months (Stress = 0.199; ANOSIM: *R* = 0.133, *P* = 0.009; Fig. 5C). However, the LEfSe verified that there was no significant difference in the species composition of the litter-inhabiting myxomycetes between six sampling months.

### Relationship between myxomycete species and environmental factors

**Figure 6:**
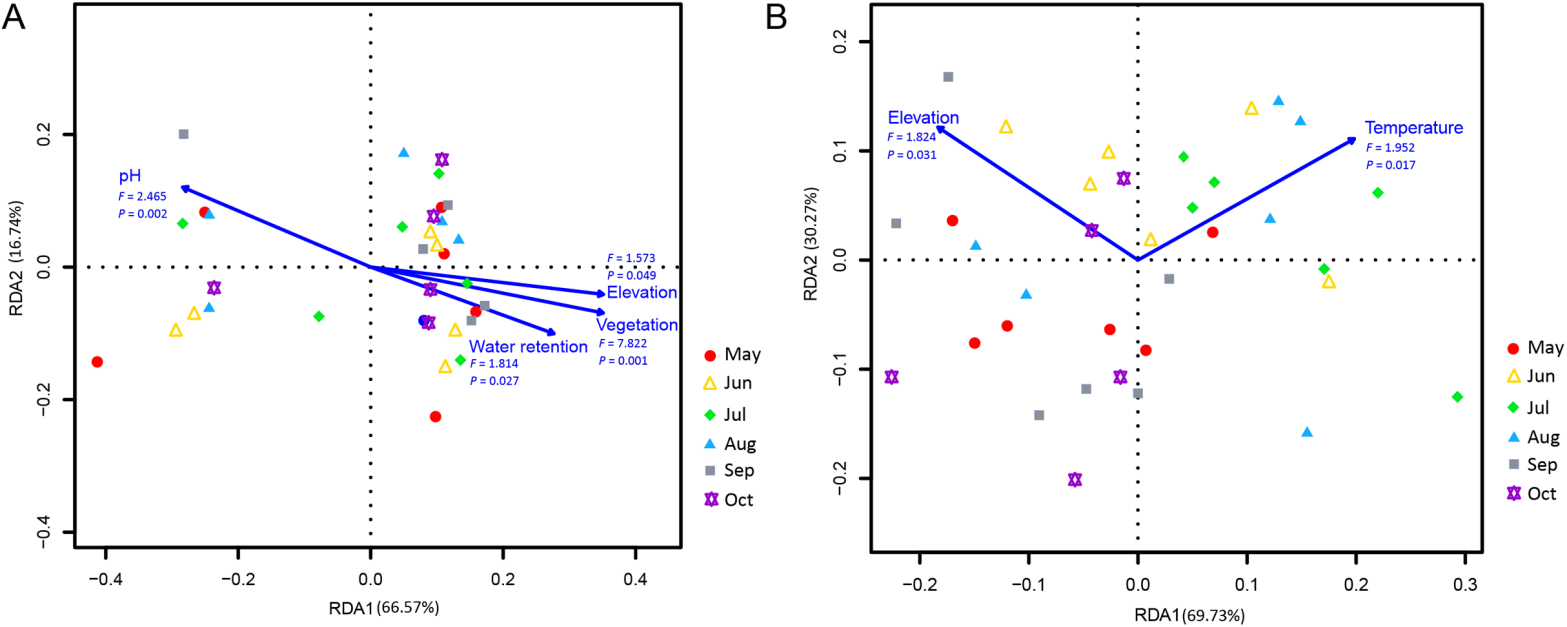
Redundancy analysis (RDA) of the relationship between the myxomycete species of (A) MC-bark and (B) MC-litter with environmental factors. Correlation between environmental variables is represented by the length and angle of arrows.

Correlations of the myxomycete species that were cultured on the MC-bark and MC-litter with environmental factors were detected using an RDA analysis. Among the total environmental variance of MC-bark assemblage, 33.43% was explained in the RDA axis1 (66.57%) and axis 2 (16.74%; Fig. 6A). Vegetation (*P* = 0.001), pH (*P* = 0.002), water retention (*P* = 0.027), and elevation (*P* = 0.049) were the main impacting factors that determined the distributions of myxomycete species on the bark. In addition, the results for MC-litter assemblage (Fig. 6B) demonstrated a significant model with elevation (*P* = 0.031) and temperature (*P* = 0.017), with 69.73% and 30.27% by the first and second axes, respectively, explained 10.50% of the cumulative variance in the weighted averages of the litter-inhabiting myxomycetes.

## Discussion

This study entailed 12 field surveys from May to October in 2015 and 2016 in the Tiantangzhai National Forest Park, Anhui Province, a subtropical region of China. A total of 73 species of myxomycetes were obtained by field collections and moist chamber cultures. The four species with the highest relative abundance, *C. oculatum*, *A. cinerea*, *C. debaryanum*, and *E. minutum*, comprised 50.29% of the total and are also widely distributed throughout the world. There were also some unusual species, such as *A. atra*, *Trichia erecta*, and *R. lycoperdon*, that had not previously been reported in subtropical forests in China. Some species, such as *Arcyria major*, *Diachea splendens*, *D. subsessilis*, *Licea castanea*, *Licea variabilis*, *P. lateritium*, *P. penetrale*, and *Trichia lutescens*, which were more common in temperate forests, had only been reported once in subtropical forests in China. Based on the distribution of myxomycetes, most of the myxomycete species recorded in the Tiantangzhai National Forest Park were diffusely distributed worldwide (Table 1), and the myxomycete species in subtropical regions strongly resembly those in tropical and temperate regions. However, it is very likely that the study area was close to the Qinling Mountains-Huaihe River Line, which is the boundary between temperate and subtropical climates in China. Therefore, from the perspective of species, many species have been reported in temperate forests many times (Stephenson, 1989; Takahashi, 2014), but only some species, such as *Physarum roseum* and *Physarum nucleatum*, were more common in the tropics (Rojas & Stephenson, 2007; Rojas, Stephenson & Huxel, 2011).

Previous research on the myxomycete species in subtropical areas include Big Bend National Park in USA (71 species, 21 genera; Ndiritu, Spiegel & Stephenson, 2009) and Thai Nguyen City in northern Vietnam (54 species,17 genera; Redeña-Santos et al., 2018). The Tiantangzhai National Forest Park had 28 and 27 mutual species with these two areas, respectively, and the Sørensen’s coefficient of community index was 0.39 and 0.43, respectively. This may be owing to geographical separation, which made the assemblage of myxomycetes different even in each habitat type, such as a subtropical forest. In the subtropical area of China, the Tiantangzhai National Forest Park shared 44 species with the Baotianman National Nature Reserve in Henan Province (which was 443 km away; 71 species, 24 genera; Gao et al., 2018) and shared 43 species with Badagongshan National Nature Reserve in Hunan Province (which was 600 km away; 91 species, 25 genera; Ukkola, Härkönen & Zeng, 2001; Härkönen, Ukkola & Zeng, 2004), and the Sørensen’s coefficient of community index was 0.61 and 0.52, respectively. It could be hypothesized that the geographical distance may affect the similarity of myxomycete species, because factors such as vegetation, temperature, and precipitation may be more similar in closer distances.

Although the ANOVA (Figs. 2B, 2C) and RDA (Fig. 6) models showed that elevation had some degree of influence on the MC-bark and MC-litter, this influence may be more closely related to the distribution of vegetation patches from a biological perspective. Vegetation with the characteristics of vertical distribution affected by elevations was one of the key environmental variables that affected the diversity of myxomycetes (Schnittler & Stephenson, 2000; Ndiritu, Spiegel & Stephenson, 2009). In deciduous broad-leaved forests and coniferous broad-leaved mixed forests, the species richness and diversity of myxomycetes were higher than those in coniferous forests, which was consistent with the results of Liu et al. (2013) and Novozhilov et al. (2017). Although these six study sites did not completely cover the area of entire forest park, they included the main forest types and tree species of this forest park. The broad-leaved and coniferous forests had the least number of shared species, indicating that the similarity of vegetation communities also affected the similarity of myxomycete species. For example, some species of Physarales, such as *Didymium clavus*, *Didymium melanospermum*, *P. bivalve*, and *P. lateritium*, only occurred in broad-leaved forests, while the relative abundance of some species of *Licea* and *Trichia* was higher in coniferous forests.

The distribution of myxomycetes on bark was susceptible to the variables of microenvironment, such as pH, texture, water retention and age of the tree (Stephenson, Schnittler & Lado, 2004). An RDA indicated that the pH and water retention of bark had significant effects on the myxomycetes. Furthermore, the LDA score of the MC-bark assemblage proved that *D. effusum* was more abundant in mixed forests, while *C. oculatum* and *C. debaryanum* were more likely to be found in coniferous forests that were more acidic (Fig. 5E). These results also confirmed the results of previous studies that most members of Stemonitidales and Echinosteliales seemed to prefer acidic conditions compared with species of Physarales (Ndiritu, Spiegel & Stephenson, 2009; Yatsiuk Iryna, Leontyev Dmitry & Shlakhter Mykhailo, 2018). Snell & Keller (2003) suggested that the occurrence and abundance of certain species were significantly related to the pH of the bark, but not to the water retention. However, Stephenson (1989) and Schnittler, Unterseher & Tesmer, 2006 suggested that the water retention of bark was positively correlated with the species of myxomycetes. This contradiction was probably caused by the increased water retention of epiphytes that were attached to the bark surface, or the difference in the thickness of the bark when sampling.

Furthermore, different temporal patterns of myxomycete diversity were obtained by comparing the two methods. July was the month with the highest incidence of myxomycete species collected in the field. However, the highest myxomycete diversity of MC-bark and MC-litter was in August and October, indicating the accumulation of propagules in the substrate. Therefore, we believe that summer was the season when fruiting bodies occur intensively, and the increased propagules in these substrates may have originated from the attachment of new spores. With the decrease in temperature and precipitation, the fruiting bodies of myxomycetes in the field decreased, but the number of propagules accumulated by the species increased. The RDA analysis indicated that temperature was a climatic factor that significantly affected the litter-inhabiting myxomycetes. Litter is the main source of forest soil organic carbon (Berg et al., 2001; Rojas & Stephenson, 2017). The change in surface properties of litter and the rate of decomposition were greatly affected by temperature (Kirschbaum, 2006). Takahashi & Hada (2012) also found that the occurrence of foliicolous myxomycetes was significantly related to temperature, and the most abundant myxomycetes were observed in Japanese temperate forests in July.

The combination of field collections and moist chamber cultures was still the most effective and reliable method to study the richness and diversity of myxomycete species. The specimens collected in the field reflect the fact that myxomycetes that complete their life history and produce fruiting bodies in a natural state, while the moist chamber culture more represents the propagules, such as spores, microcysts, and sclerotia on substrates (Martin & Alexopoulos, 1969). Therefore, the combination of field collections and moist chamber cultures can more completely reflect the diversity of myxomycetes in the ecosystem. Only nine shared species indicated a difference in the composition of myxomycetes species between the two methods (Table 1). Some species made these two methods irreplaceable and complementary. Myxomycetes that had a large size or bright colors were more conspicuous when collecting fruiting bodies in the field (Schnittler & Stephenson, 2002), including some species of *Lycogala*, *Tubifera*, and *Enteridium*. However, species with minute fruiting bodies such as *Clastoderma*, *Echinostelium*, and *Licea*, could be more easily observed on the substrate of cultures. However, with the increasing availability of genetic information, traditional myxomycete taxonomy is being increasingly challenged (Walker & Stephenson, 2016). For example, a morphological species was represented by several ribotypes and cryptic speciation, as shown in the details of *Tricia varia* (Feng & Schnittler, 2015), which may be the rule rather than an exception. However, owing to the late start of molecular biology of myxomycetes and the lack of universal primers, the true diversity of myxomycetes has not been revealed.

## Conclusions

This study provided insights into the species composition, spatiotemporal dynamic change, and drivers of myxomycete communities in subtropical forests of China, which was of substantial significance to make up the gap of myxomycetes species in the relatively insufficiently studied subtropical forests. A total of 73 species indicated the abundant myxomycete resources of Tiantangzhai National Forest Park, among which 37 species were collected in the field, and 38 and 37 species were obtained from bark and litter culture in moist chambers, respectively. The myxomycete species obtained by different experimental methods exhibited different temporal and spatial dynamics. For example, the sampling months had more significant impacts on field collected and litter-inhabiting myxomycetes than the types of forests. Temperature was the main factor that affected the litter-inhabiting myxomycetes. The species richness and diversity of bark-inhabiting myxomycetes in coniferous and broad-leaved mixed forests were significantly higher than those of coniferous forests, among which vegetation, pH, water retention, and elevation were the primary factors.

## Supplemental Information

10.7717/peerj.12059/supp-1 Supplemental Information 1 Specimen records of myxomycetes in Tiantiangzhai National Forest Park.

10.7717/peerj.12059/supp-2 Supplemental Information 2 Relative abundance of myxomycetes divided by forest type (DBF: deciduous broad-leaved forest; CBMF: coniferous broad-leaved mixed forest; CF: coniferous forest) and month (May: May; Jun: June; Jul: July; Aug: August; Sep: September; Oct: October).

10.7717/peerj.12059/supp-3 Supplemental Information 3 ANOVA results of species richness (S) and Shannon diversity (H′) of myxomycetes obtained from samples of bark and litter with the moist chamber culture technique, and field collections analyzed for variation among sampling sites (QF, JP, YF, PP, WF, OB).

10.7717/peerj.12059/supp-4 Supplemental Information 4 Records of myxomycete species collected in the field in different months (May: May; Jun: June; Jul: July; Aug: August; Sep: September; Oct: October; Study sites: QF, JP, YF, PP, WF, OB).

10.7717/peerj.12059/supp-5 Supplemental Information 5 Records of myxomycete species of MC-bark in different forest types (DBF: deciduous broad-leaved forest; CBMF: coniferous broad-leaved mixed forest; CF: coniferous forest; Study sites: QF, JP, YF, PP, WF, OB; Repetitive sampling plots: A–E).

10.7717/peerj.12059/supp-6 Supplemental Information 6 Records of myxomycete species of MC-litter in different months (May: May; Jun: June; Jul: July; Aug: August; Sep: September; Oct: October; Study sites: QF, JP, YF, PP, WF, OB).

10.7717/peerj.12059/supp-7 Supplemental Information 7 Results of ANOSIM in different assemblages based on Bray-Curtis distance (May: May; Jun: June; Jul: July; Aug: August; Sep: September; Oct: October; DBF: deciduous broad-leaved forest; CBMF: coniferous broad-leaved mixed forest; CF: coniferous forest).

10.7717/peerj.12059/supp-8 Supplemental Information 8 Biomarkers with significant differences in LEfSe of myxomycete species collected in the field in different sampling months.

10.7717/peerj.12059/supp-9 Supplemental Information 9 Biomarkers with significant differences in LEfSe of myxomycete species of MC-bark in different forest types (DBF: deciduous broad-leaved forest; CBMF: coniferous broad-leaved mixed forest; CF: coniferous forest).

10.7717/peerj.12059/supp-10 Supplemental Information 10 Biomarkers with significant differences in LEfSe of myxomycete species of MC-litter in different sampling months.

10.7717/peerj.12059/supp-11 Supplemental Information 11 Records of myxomycete species of MC-bark in different months (May: May; Jun: June; Jul: July; Aug: August; Sep: September; Oct: October; Sampling sites: QF, JP, YF, PP, WF, OB).

## Additional Information and Declarations

### Field Study Permissions

The following information was supplied relating to field study approvals (*i.e*., approving body and any reference numbers): The research was conducted in Tiantangzhai National Forest Park. Our experimental sites are located at the edge of the tourism area of Tiantangzhai National Forest Park. When we collected samples, we showed our identity and intention to the park staff, and then we got their consent and help. Therefore, approval documents were not required.

### Data Availability

The following information was supplied regarding data availability: The raw data are available in the Supplemental Files.
